# A Mini-Review of Flavone Isomers Apigenin and Genistein in Prostate Cancer Treatment

**DOI:** 10.3389/fphar.2022.851589

**Published:** 2022-03-11

**Authors:** Xiaozhen Ji, Kai Liu, Qingyue Li, Qun Shen, Fangxuan Han, Qingmei Ye, Caijuan Zheng

**Affiliations:** ^1^ Hainan General Hospital and Hainan Affiliated Hospital of Hainan Medical University, Haikou, China; ^2^ Key Laboratory of Tropical Medicinal Plant Chemistry of Ministry of Education, College of Chemistry and Chemical Engineering, Hainan Normal University, Haikou, China; ^3^ Key Laboratory of Tropical Medicinal Plant Chemistry of Hainan Province, College of Chemistry and Chemical Engineering, Hainan Normal University, Haikou, China

**Keywords:** prostate cancer, resistance, flavone, apigenin, genistein

## Abstract

The initial responses to standard chemotherapies among prostate cancer (PCa) patients are usually significant, while most of them will finally develop drug resistance, rendering them with limited therapies. To discover new regimens for the treatment of PCa including resistant PCa, natural products, the richest source of bioactive compounds, can serve as a library for screening and identifying promising candidates, and flavones such as apigenin and genistein have been used in lab and clinical trials for treating PCa over decades. In this mini-review, we take a look into the progress of apigenin and genistein, which are isomers, in treating PCa in the past decade. While possessing very similar structure, these two isomers can both target the same signaling pathways; they also are found to work differently in PCa cells. Given that more combinations are being developed and tested, genistein appears to be the more promising option to be approved. The anticancer efficacies of these two flavones can be confirmed by *in-vitro* and *in-vivo* studies, and their applications remain to be validated in clinical trials. Information gained in this work may provide important information for new drug development and the potential application of apigenin and genistein in treating PCa.

## Introduction

Prostate cancer (PCa) is the most commonly diagnosed cancer and the second leading cause of cancer death in men (do Pazo and Webster, 2021; Whitaker et al., 2020; Komura et al., 2018; Dunn, 2017). There will be an estimate of 248,530 cases and 34,130 deaths in the United States in 2021, posing a serious burden on health care in the United States and worldwide. The initial response rate of androgen deprivation therapy is usually high, while a significant proportion (around 90%) of PCa patients develops cartration-resistant PCa (CRPC) (Thoma, 2016; Huang et al., 2018; Makino et al., 2021; Welti et al., 2021). And even worse, the vast majority of CRPC patients will eventually become resistant to the first-line treatment docetaxel and enzalutamide (Scott, 2018; Lin et al., 2020; Moussa et al., 2020; Peery et al., 2020). In addition, death is usually not caused by the primary tumor but by the formation of distinct metastatic tumors that show resistant properties to varied therapies (Shore, 2020; Sayegh et al., 2021). There are unmet clinical needs to tackle drug resistance in PCa through discovering novel chemical entities or certain combinations.

**FIGURE 1:**
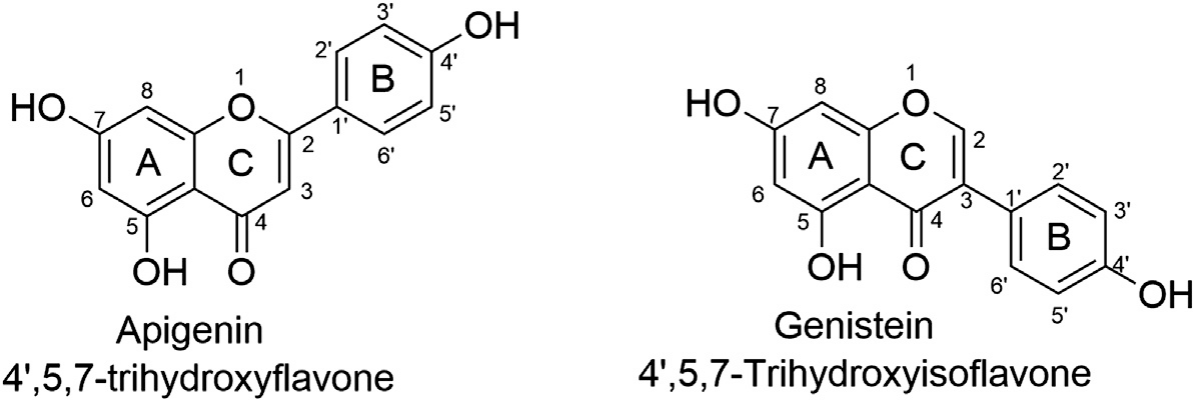
The chemical structures of isomers apigenin and genistein. Apigenin and genistein share the same formulate but structurally differ at the linking position of ring B and C. Such minor difference has led to significant different pharmacological profiles.

Natural active products serve as a rich resource for the identification of hit compounds that can be used for the consequent structural modification and optimization, accounting for approximately 40% of FDA-approved drugs (Cui et al., 2018; Newman and Cragg, 2020). Among all those various active components found in traditional herbals, flavone is one of the most studied both in lab and in clinical trials (Amawi et al., 2017; Bondonno et al., 2019; Bisol et al., 2020). In addition, of roughly 3,000 flavonoids (Ye et al., 2019), apigenin and genistein, two isomers as shown in Figure 1, are particularly interesting to us.

Apigenin, 4′, 5, 7-trihydroxyflavone, is abundant in vegetables and certain foods that have been used as medicinal plants worldwide for centuries (Salehi et al., 2019; Su et al., 2020; Xu et al., 2021). Genistein, 4′, 5, 7-trihydroxyisoflavone, was first reported in 1899, and its structure was identified in 1926 (Jung et al., 2020). Apigenin and genistein both target dozens of pharmacological targets, such as estrogen receptors, ABC transporters, membrane proteins, mitochondria-associated proteins, cell cycle-associated proteins, epigenetic regulators, cytokines, and many signaling pathways including NF-κB, MAPK/ERK, JAK/STAT, PI3K/Akt and Wnt/β-catenin pathways, exhibiting therapeutic applications in cardiovascular diseases, neurodegenerative diseases, cancers, etc. (Mukund et al., 2017; Jaiswal et al., 2019; Kim and Park, 2020; Javed et al., 2021). Apigenin and genistein have been launched into market as dietary supplements for decades; meanwhile, they are also actively tested in clinical trials, especial for genistein as more clinical trials use it as a candidate for the treatment of various diseases including malignant cancers (Yu et al., 2021). Thus, in the current mini-review, we attempted to have an overview of the progress made in the past decade of the studies (2012–2021) of apigenin and genistein in the treatment of PCa, including resistant forms of PCa, as flavonoids have also shown promising therapeutic application in resistant cancers (Ye et al., 2019). We would like to have a brief discussion of the differences except their structures, including acting modes, targets, and the signaling pathways network.

## Apigenin and Genistein Show Great Potentials in Treating PCa

One thing we need to bear in mind is that these two flavones are multi-targeted or multi-functional compounds, meaning they exert their anticancer activities via multiple mechanisms. We attempted to interpret the pharmacological effects including inhibiting cancer cells proliferation and metastasis, enhancing the sensitivity of certain chemotherapeutics.

## Apigenin

### Inhibiting IκB Kinase α (IKKα)

IKKα functions to activate NF-κB that works as a critical mediator to regulate the crosstalk of inflammation and cancer initiation and progression, serving as a druggable target (Lv et al., 2020; Mo et al., 2021).

Shukla et al. (2015) confirmed that apigenin could directly bind IKKα. Apigenin (2.5–20 μM) attenuated IKKα kinase activity and suppresses the activation of NF-κB/p65 in human PCa PC-3 and 22Rv1 cells. Apigenin caused cell cycle arrest similar to the effects induced by the knockdown of IKKα. *In-vivo* studies of xenograft mouse model indicate that apigenin (20 and 50 μg/day, gavage) suppressed tumor growth, lowered cancer cells proliferation, and enhanced apoptosis, mediated with the inhibition of p-IKKα, NF-κB/p65 (Shukla et al., 2015). This study suggested that apigenin inhibited cancer growth via suppressing IKKα and its downstream targets NF-κB (Shukla et al., 2015).

In the same group, Shukla et al. (2014a) identified another mechanism related to IKKα/β in apigenin-induced cytotoxicity in PCa (Shukla et al., 2014a). Forkhead box O (FoxO) transcription factors play an important role as tumor suppressors in human malignancies (Yadav et al., 2018). Disruption of FoxO activity due to loss of phosphatase and tensin homolog and the activation of phosphatidylinositol-3 kinase (PI3K)/Akt are frequently observed in PCa. In a model of TRAMP (transgenic adenocarcinoma of the mouse prostate) mice, Apigenin (20 and 50 μg/day, 6 days/week for 20 weeks) treatment suppressed the tumor growth and metastasis (Shukla et al., 2014a). Apigenin, via increasing nuclear retention and decreasing the binding of FoxO3a with 14-3-3, reduced the p-Akt (Ser473) and FoxO3a (Ser253) as confirmed by histologic analyses. Similar results were also observed in human PCa LNCaP and PC-3 cells, as apigenin (10 and 20 μM) increased the binding of FoxO3a with p27/Kip1, leading to cell arrest at G1 phase. *In-silico* molecular modeling study confirmed a strong affinity of apigenin to either IKKα or IKKβ, which was then validated by *in-vitro* ELISA assay (Shukla et al., 2014a). Further study showed that apigenin can preferably bind IKKα over IKKβ, leading to the inactivation of NF-κB in PC-3 and 22Rv1 cells, providing convincing evidence that apigenin suppressed PCa progression by targeting the IKK/NF-κB pathway (Shukla et al., 2014a).

### Inhibiting Inhibitor of Apoptosis Proteins

IAP family members, including XIAP, c-IAP1/2 and survivin, are historically regarded as the major regulators of the apoptosis pathway due to their ability to inhibit pro-apoptotic caspases, highlighting that IAPs can serve as potential therapeutic targets in cancers (Peery et al., 2017; Dizdar et al., 2018; Lalaoui and Vaux, 2018; Rada et al., 2018; Hrdinka and Yabal, 2019).

Shukla et al. (2014b) found that in human PCa PC-3 and DU145 cells that are androgen-refractory, apigenin (5–40 μM) treatment decreased cell viability and induced significant apoptosis mediated by the up-regulation of cleaved caspase 3/9 and PARP (poly [ADP-ribose] polymerase), accompanied with down-regulated levels of IAP members including XIAP, c-IAP1/2, and survivin, as well as the decreased levels of Bcl-xL and Bcl-2 and the increased cytochrome C, a key player in executing apoptosis. Importantly, pretreatment of the inhibitors of caspase 9 (z-LEHD-fmk) and caspase 3 (z-DEVD-fmk) was able to alleviate apigenin-induced apoptosis. Further study indicated that the increased Bax was mediated by the inhibition of histone deacetylases (HDACs) induced by apigenin, which then caused the Ku70 acetylation that lead to the dissociation of Bax from Ku70. In addition, apigenin (20, 50 μg/day, gavage) also showed dose-dependent tumor growth inhibition in PC-3 cells xenograft mouse model, strengthening its role as an anticancer agent (Shukla et al., 2014b).

### Targeting Adenine Nucleotide Translocase-2

ANT2 is a mitochondria-associated protein that functions to exchange ADP and ATP (Jang et al., 2008; Lee et al., 2019), serving as a therapeutic target that can be regulated by apigenin but not genistein as confirmed by Oishi et al. (2013). Apigenin was able to bind and inhibit ANT2, leading to the increased death receptor 5 (DR5) and improved apoptosis induced by Apo2L/TRAIL. Meanwhile, these effects induced by apigenin could be attenuated by the ANT2 knockdown, verifying that its mechanism involved ANT2 (Oishi et al., 2013).

### Suppressing Epithelial-to-Mesenchymal Transition

Apeginin at a wide range of concentrations/doses has been found to repress cancer cells migration. EMT is a process that cancer cells initiate the migration and invasion, playing critical roles in cancer migration and drug resistance (Brabletz et al., 2018; Georgakopoulos-Soares et al., 2020).

Zhu et al. (2015) found that apigenin (10–80 μM) suppressed the proliferation of the DU145 cells via inducing G2/M phase cell cycle arrest. Aigenin (5–20 μM) inhibited the migration and invasive potentials as shown in the wound healing assay. Western blot analysis showed that upon apigenin (5, 10, 20 μM) treatment, E-cadherin, an essential component to reverse EMT, was significantly up-regulated (Zhu et al., 2015).

Later, Chien et al. (2019) found that Apigenin (0–80 μM) inhibited cell viabilities of four human PCa cell lines including metastatic hormone-sensitive cell line (LNCap) and metastatic CRPC cell lines (DU145, PC-3, and PC-3 M) in both time- and dose-dependent manners, and it reduced colony formation numbers of all 4 cell lines, suggesting it is a broad-spectrum anticancer agent. Apigenin (20 and 40 μM) significantly suppressed the characteristics of migration and invasion of PC-3 M cells. More importantly, apigenin (3 mg/kg/day, IP) significantly reduced tumor metastasis to the lung, liver, pancreas, spine, bone, and brain in an intracardiac injection model constructed by PC-3 M cells, leading to a prolonged survival rate. SPOCK1 (Sparc/osteonectin, cwcv, and kazal-like domains proteoglycan 1) is a crucial modulator in prompting EMT, which is overexpressed in PC-3 M cells. Apigenin (10, 20, 40 μM *in-vitro* and 3 mg/kg/day *in-vivo*) induced apoptosis and inhibited metastasis through down-regulating SPOCK1, as well as Snail1/2, which can be reversed by exotic overexpression of SPOCK1, suggesting a pharmacological targeting effect of apigenin (Chien et al., 2019).

### Suppressing Cancer Stem Cells

CSCs, a subset of cancer cells that show self-renewing and differentiation properties, are regarded as key players that are responsible for chemoresistance, tumor relapse, and metastasis (Sancho et al., 2016; Cui et al., 2017; Luo et al., 2018; Carvalho et al., 2021). Apigenin has been shown to negatively regulate CSCs, resulting in the re-sensitization of certain chemotherapeutics.

Erdogan et al. (2017) tested apgenin’s effect towards CD44^+^ PCa stem cells that were isolated from human androgen-independent PC-3 PCa cells. Apigenin (15 μM) significantly enhanced cisplatin’s (7.5 μM) cytotoxic and apoptosis-inducing and migration-suppressing effects through the down-regulation of anti-apoptotic Bcl-2, sharpin and survivin, and the up-regulation of pro-apoptotic members such as caspase-8, Apaf-1 (apoptotic protease activating factor-1), and p53 mRNA expression. The combined therapy down-regulated p-PI3K, p-Akt, NF-κB, and arrested cell cycle at G2/M and S phases (Erdogan et al., 2017).

A similar study by the same group (2016) showed that apigenin (0–100 μM) inhibited CSCs (CD44^+^ PCa stem cells) and PC-3 cell survival, with a significant increase of p21 and p27, both of which are anti-apoptotic proteins. It appeared that apigenin induced apoptosis via an extrinsic caspase-dependent pathway mediated by up-regulating the mRNA expressions of caspases-8, -3, and TNF-α, but failed to trigger the intrinsic pathway as determined by the levels of pro-apoptotic Bax, cytochrome C, and Apaf-1 in CSCs. While in contrast in PC-3 cells, apigenin exerted cytotoxic effects via intrinsic apoptosis pathway. Apigenin (25 μM) suppressed the migration of CSCs via down-regulating matrix metallopeptidases-2, -9, (MMP2/9), and Snail1/2. Similarly, the expressions of NF-κB p105/p50, p-PI3K, and p-Akt were all decreased after apigenin treatment. Moreover, apigenin treatment significantly reduced the protein expression of pluripotency marker Oct3/4, indicating apigenin’s strong potential in suppressing CSCs, warranting further *in-vivo* study (Erdogan et al., 2016).

## Genistein

Genistein in some cases shows similar or even the same mechanism as apigenin toward certain targets, such as CSCs (Zhang et al., 2012), PI3K/Akt signaling pathway (Song et al., 2020), MMP2 and apoptosis-related proteins including caspase 3 (Shafiee et al., 2020), while genistein appears to regulate more targets than apigenin.

### Inactivating DNA Repair Pathways

Homologous recombination (HR) and the nonhomologous end joining (NHEJ) pathways are two DNA repair pathways that are activated by PCa cells when treated by certain chemotherapeutics, resulting in drug resistance (Sharda et al., 2020; Xiang et al., 2020; Filsinger et al., 2021). Genistein can work as chemotherapy sensitizer via impacting HR and NHEJ. Tang et al. (2018) developed a triple combination therapy composed with genistein (30 μM), AG1024 (10 μM), and radiotherapy. As shown in PC-3 and DU145 cells, the pretreatment of genistein and AG1024 could markedly enhance the inhibition of cells proliferation and induction of apoptosis induced by radiotherapy, which was mediated by the up-regulated Bax and cleaved caspase 3, leading to reduced colony formation (Tang et al., 2018). The triple combination induced a cell arrest at the S phase and decreased the G2/M phases. Further study indicated that this combination could induce double-strand breaks mediated by the inactivation of HR and NHEJ pathways, evidenced by the down-regulation of DNA-PKcs (Thr2609), Rad51, and Ku70. The *in-vivo* study also confirmed that this triple combination (100 mg/kg/day of genistein and AG1024, plus X-irradiation) surpassed the efficacies of other dual therapies, without showing any obvious adverse effects (Tang et al., 2018).

Genistein was also shown to regulate another key player in DNA repair, which was the APE1 (apurinic/apyrimidinic endonuclease1) that served as an oxidative DNA repair enzyme (Maayah et al., 2015; Pekhale et al., 2017; Lebedeva et al., 2020). In order to reduce the severely toxic effects toward normal tissues of doxorubicin (DOX), a nanoparticles system named as DOX-NPs, loaded with DOX and genistein, was synthesized by Wang et al. (2018) and evaluated *in-vitro* and *in-vivo*. Not only could genistein, when worked as an APE1 inhibitor, enhance DOX-induced cell death mediated by overproduced ROS (reactive oxygen species), it can also alleviate DOX-induced toxic effects. DOX-NPs showed a strong activity in suppressing distant metastasis in the *in-vivo* model (Wang et al., 2018).

### Inhibiting Glucose Transporter 1

Glut-1 functions to the transportation of glucose across cell membrane, facilitating ATP production (Almahmoud et al., 2019; Pragallapati and Manyam, 2019; Qamar et al., 2019; Ahopelto et al., 2020). Enhanced Glut-1 protein as well as COX-2 (cyclooxygenase-2) is reported as one of the major driving factors, leading to the initiation and progress of PCa (Carreño et al., 2019; Gasinska et al., 2020; King et al., 2020; Joshi et al., 2021). Tian et al. (2019) synthesized a liposomal system composed with COX-2 inhibitor celecoxib and genistein (100 μM celecoxib and 10 μM genistein), and this liposomal system exhibited selective cytotoxic effects towards PC-3 and LNCaP cells over non-cancer cells via inducing apoptosis as confirmed by up-regulated cleaved caspase 3, and it suppressed PC-3 cells migration. This liposomal system could induce ROS production, decrease GSH level, and inhibit COX-2 and Glut-1 receptors simultaneously, leading to decreased glucose uptake in PC-3 and LNCaP cells, indicating a dual targeting (Tian et al., 2019). Further models are clearly needed to elucidate its safety and efficacy.

### Demethylating Effect

Similar as apigenin, genistein can also regulate epigenetic proteins. Methylation of certain genes can be hijacked by cancer cells to promote its tumorigenesis (Wu et al., 2020; Zhao et al., 2020; Tolkach et al., 2021), while genistein was shown to down-regulate methylation.

ER-β (Estrogen receptor β) promoter hyper-methylation has a tumor inducing effect in PCa. Mahmoud et al. (2015) found that genistein (0.5–10 μM) significantly reduced the methylation of ER-β promoter accompanied with corresponding dose-dependent increased of ER-β expression in LNCaP and LAPC-4 but not in PC-3 cells with lower basal levels of ER-β promoter methylation. Genistein (0.5–10 μM) could down-regulate DNA methyl transferases (DNMTs) and increase the phosphorylation, nuclear translocation, and transcriptional activity of ER-β (working as a cancer suppressor) in all three PCa cell lines. Inhibitory effects of genistein on LAPC-4 and PC-3 cell proliferation were diminished using a specific ER-β antagonist, suggesting an ER-β-mediated mechanism (Mahmoud et al., 2015).

### Inhibiting Insulin-like Growth Factor-1 Receptor

IGF-1, an essential player in anabolism, is found to be elevated in several different cancers, including PCa (Uzunlulu et al., 2016; Mancarella et al., 2017; Larsson et al., 2020; Ohishi et al., 2020). The inhibition of IGF-1 and its downstream signaling pathways may have therapeutic implications (Nordstrand et al., 2013; Nordstrand et al., 2017).

Lee at al. (2012) found that genistein treatment caused a significant inhibition of IGF-1-stimulated PC-3 cells growth through decreasing the proliferation of IGF-1-stimulated cells and inducing cell arrest at G0/G1 phase (Lee et al., 2012). Genistein effectively inhibited the phosphorylation of IGF-1R and the phosphorylation of its downstream targets, such as Src, Akt, and GSK-3β (glycogen synthase kinase-3β), and it greatly attenuated IGF-1-induced β-catenin signaling and cyclin D1 levels in PC-3 cells (Phillip et al., 2012).

### Modulating miRNAs and Long Non-coding RNAs

Different with apigenin, genistein could regulate miRNAz and lncRNAs to achieve its treatment efficacies in PCa.

Hirata et al. (2014) found that miRNA-1260b expression was significantly decreased by genistein (25 μM for 4 days) in PC-3 and DU-145 cells; meanwhile, the knockdown of miR-1260b suppressed cell proliferation, invasion, migration, and TCF reporter activity in PC cells, similar phenomenon as genistein treatment (Hirata et al., 2014).

Chiyomaru et al. (2013a) found that genistein may impact the axis of lncRNAs and miRNAs as confirmed by microarray assay which showed that lncRNA HOTAIR and miR-34a were regulated by genistein (25 µM) in PC-3 and DU145 cells (Chiyomaru et al., 2013a). By this same group (2013), they reported another finding that genistein (25 and 50 µM) might up-regulate tumor suppressor miR-574-3p in PC-3 cells and in PCa tissues (Chiyomaru et al., 2013b). In addition, Chiyomaru et al. (2012) also identified another key player, oncogenic miR-151, which can be down-regulated by genistein (25 µM) in PC-3 and DU145 cells (Chiyomaru et al., 2012).

### Certain Combinations That Induced Apoptosis

Genistein could work as a chemosensitizer.

Zhang et al. (2013) found that genistein (5 and 10 μg/ml) when combined with cabazitaxel (5–100 nM) could enhance the sensitivity of cabazitaxel to metastatic CRPC cells *in-vitro* and *in-vivo* via increasing the expression of pro-apoptotic Bax and cleaved caspase 3 (Zhang et al., 2013). In a PC-3-luciferase xenograft model, the combined treatment with genistein (100 mg/kg) and cabazitaxel (5 mg/kg) significantly retarded the growth of mCRPC when compared to vehicle control, cabazitaxel, or genistein (Zhang et al., 2013).

Another study showed that the combination of topotecan and genistein exhibited better efficacy in LNCaP and PCa cells than either monotherapy, which was mediated by inducing apoptosis activated by caspase-3 and -9 and by ROS generation (Hörmann et al., 2012).

Another genistein nano-suspension (named as BIO 300) was synthesized by Jackson et al. (2019). BIO 300 positively synergized with radiation, resulting in tumor growth delay and prolonged survival as confirmed in hormone-sensitive and insensitive prostate tumor-bearing mice (Jackson et al., 2019). Their data also strongly support the clinical translation of BIO 300 for mitigation of ED (erectile dysfunction) among PCa patients (Jackson et al., 2019).

## Clinical Evidence

Many ongoing clinical studies of different stages have also revealed that genistein may benefit PCa patients via either inhibiting metastasis or suppressing tumor growth (Pavese et al., 2014). Retrospective clinical studies have shown that high consumption of soybean products (genistein as its major active component) has been associated with a low incidence of PCa (Hwang et al., 2009). Wu et al. (2015) reported a preliminarily evaluation of the associations among plasma genistein and PCa in a Chinese population composed with 100 men aged over 40 and diagnosed with PCa (Zhu et al., 2015) or PCa-free (Zhang et al., 2012), and analyzed the physiological data before and after certain doses of genistein dietary intake. Their data showed that cancer-free patients possessed higher plasma genistein concentration than PCa patients, and the plasma genistein concentration negatively correlated to PCa (Wu et al., 2015). Bilir et al. (2017) invested the effects of genistein on Norwegian patients who received 30 mg genistein or placebo capsules daily for 3–6 weeks before prostatectomy. Microarrays and qPCR data showed that several differentially methylated sites and expressed genes between placebo and genistein groups. Importantly, the MYC activity reduced and PTEN activity increased in patients receiving genistein, highlighting the Pca-preventive of genistein (Bilir et al., 2017).

While some clinical studies showed unfavorable results towards the remission of PCa (Jarrard et al., 2016), or the plasma genistein concentration was not associated with PCa risk in large cohort of European men (Travis et al., 2012), more clinical trials with well-filtered qualified PCa patients are warranted.

## Summary and Brief Discussion

The above information strongly supports the potential application of apigenin and genistein in treating PCa including resistant forms of PCa as shown in Table 1.

**TABLE 1:** Summary (2012–2021) of apigenin and genistein in the treatment of PCa.

Flavone	Mechanisms	*In-vitro*	*In-vivo*	References
Apigenin	Inhibiting IKKα	Inhibiting the proliferation of PC-3 and 22Rv1 cells	Reducing tumor growth of PC-3 and 22Rv1 cells xenografts	Shukla et al. (2015)
	Targeting PI3K/Akt/FoxO	Inducing cell arrest of PC-3 and 22Rv1 cells	Reducing tumor growth and metastasis of TRAMP mice	Shukla et al. (2014a)
	Inhibiting IAP	Inducing apoptosis of PC-3 and DU145 cells	Reducing tumor growth of PC-3 cells xenograft	Shukla et al. (2014b)
	Inhibiting ANT2	Sensitizing TRAIL in DU145 cells	ND^a^	Oishi et al. (2013)
	Suppressing EMT	Inhibiting DU145 and PC-3 M cells proliferation and migration	Reducing tumor growth and metastasis of PC-3 M cells xenograft	Zhu et al., 2015; Chien et al., 2019
	Suppressing CSCs	Sensitizing cisplatin in CD44^+^ PCa stem cells	ND	Erdogan et al. (2016); Erdogan et al. (2017)
Genistein				
	Suppressing CSCs	Inhibiting the tumorigenicity of PCa CSCs	Reducing tumor growth of PCa CSCs cells xenograft	Zhang et al. (2012)
	Targeting PI3K/Akt	Inhibiting PC-3 cells proliferation and migration	Reducing tumor growth of PC-3 cells xenograft	Song et al. (2020)
	Targeting MMP2 and apoptosis	Inhibiting PC-3 cells proliferation and migration	ND	Shafiee et al. (2020)
	Targeting DNA repair	Sensitizing AG1024 and radiotherapy in PC-3 and DU145 cells	Reducing tumor growth of DU145 cells xenograft	Tang et al. (2018)
		Sensitizing DOX	Reducing tumor growth	Wang et al. (2018)
	Inhibiting Glu-1	Synergizing with celecoxib in PC-3 and LNCap cells	ND	Tian et al. (2019)
	Demethylating	Inhibiting LNCap and LAPC cells proliferation	ND	Mahmoud et al. (2015)
	Inhibiting IGF-1	Inhibiting PC-3 cells growth	ND	Lee et al. (2012)
	Regulating miRNA and lncRNA	Inhibiting PC-3 and DU145 cells	ND	Chiyomaru et al. (2012); Chiyomaru et al. (2013b)
	Inducing apoptosis	Sensitizing cabazitaxel, topotecan and radiotherapy in PC-3 or LNCaP cells	Reducing tumor growth of PC-3 cells xenograft	Hörmann et al., 2012; Zhang et al. (2013); Jackson et al. (2019)

a ND, not determined.

**FIGURE 2:**
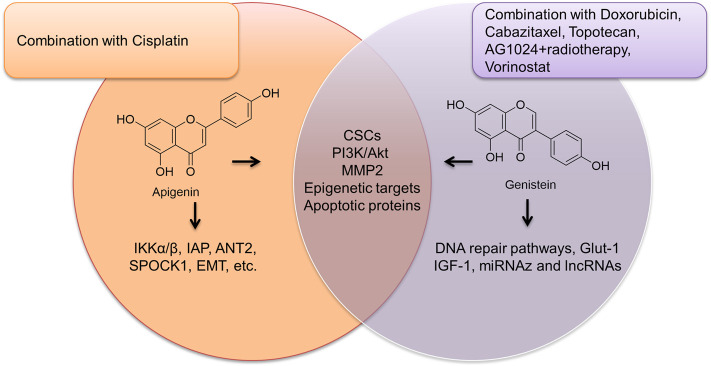
The overview of apigenin and genistein in PCa treatment.

While apigenin and genistein are isomers, they appear to treat PCa via a slight different mechanism. Both of them can regulate CSCs (Zhang et al., 2012; Erdogan et al., 2016), PI3K/Akt (Shukla et al., 2014a; Song et al., 2020), MMP2, and apoptosis-related proteins including caspase 3 (Shafiee et al., 2020) and DNA damage-associated repair pathway (Phillip et al., 2012; Sharma et al., 2014; Tang et al., 2018). While apigenin was able to inhibit IAP members, genistein seems to impact membrane-associated proteins, such as certain receptors including Glut-1 and IGF-1 (Lee et al., 2012; Tian et al., 2019) as summarized in Figure 2.

Genistein appears to be more useful in combination than apigenin that only was shown to sensitize cisplatin. Several combinations regimens, such as genistein + celecoxib (Tian et al., 2019), genistein + cabazitaxel (Zhang et al., 2013), genistein + topotecan (Hörmann et al., 2012), genistein + vorinostat, as well as triple combination of genistein + AG1024 + radiotherapy (Jackson et al., 2019), were optimized and tested in cell-based assay and in animal models, strongly supporting its potential as chemotherapy in PCa.

Furthermore, these two flavones can serve as leading compound that undergo structural modifications to achieve higher activities but lower toxic effects (Chen et al., 2015; Xiong et al., 2015; George et al., 2018).

## Conclusion

Flavones isomers apigenin and genistein, by mono-therapy or combinational therapy, exhibited great potentials in the treatment of PCa including resistant PCa.
